# MRI-based radiomics model and nomogram for predicting the outcome of locoregional treatment in patients with hepatocellular carcinoma

**DOI:** 10.1186/s12880-023-01030-5

**Published:** 2023-05-30

**Authors:** Yuxin Wang, Zhenhao Liu, Hui Xu, Dawei Yang, Jiahui Jiang, Himeko Asayo, Zhenghan Yang

**Affiliations:** 1 Department of Radiology, Beijing Friendship Hospital, Capital Medical University, Beijing, 100050 China; 2 Department of Radiology, Affiliated Hospital of Changzhi Institute of Traditional Chinese Medicine, Changzhi, 046099 China

**Keywords:** Hepatocellular carcinoma, Radiomics, Magnetic resonance imaging, Locoregional treatment

## Abstract

**Background:**

Prediction of locoregional treatment response is important for further therapeutic strategy in patients with hepatocellular carcinoma. This study aimed to investigate the role of MRI-based radiomics and nomogram for predicting the outcome of locoregional treatment in patients with hepatocellular carcinoma.

**Methods:**

The initial postoperative MRI after locoregional treatment in 100 patients with hepatocellular carcinoma was retrospectively analysed. The outcome was evaluated according to mRECIST at 6 months. We delineated the tumour volume of interest on arterial phase, portal venous phase and T2WI. The radiomics features were selected by using the independent sample t test or nonparametric Mann‒Whitney U test and the least absolute shrinkage and selection operator. The clinical variables were selected by using univariate analysis and multivariate analysis. The radiomics model and combined model were constructed via multivariate logistic regression analysis. A nomogram was constructed that incorporated the Rad score and selected clinical variables.

**Results:**

Fifty patients had an objective response, and fifty patients had a nonresponse. Nine radiomics features in the arterial phase were selected, but none of the portal venous phase or T2WI radiomics features were predictive of the treatment response. The best radiomics model showed an AUC of 0.833. Two clinical variables (hCRP and therapy method) were selected. The AUC of the combined model was 0.867. There was no significant difference in the AUC between the combined model and the best radiomics model (*P* = 0.573). Decision curve analysis demonstrated the nomogram has satisfactory predictive value.

**Conclusions:**

MRI-based radiomics analysis may serve as a promising and noninvasive tool to predict outcome of locoregional treatment in HCC patients, which will facilitate the individualized follow-up and further therapeutic strategies guidance.

## Background

Hepatocellular carcinoma (HCC) is the fourth leading cause of death from cancer and there are around 841,080 new cases every year [1, 2], which places a heavy burden on the family economy and social health care. Unfortunately, up to 70% of patients already have advanced HCC at the time of their first visit, so they have lost the opportunity for possible radical treatment such as resection and transplantation [3]. As an important method of locoregional treatment (LRT), transcatheter arterial chemoembolization (TACE) is not only the most common treatment for intermediate stage HCC but is also the first-line therapy across disease stages in China, North America and Europe [4]. TACE even acts as a bridging or downstaging method before resection and transplantation [5]. In addition, according to the Barcelona Clinical Liver Cancer (BCLC) staging system, radiofrequency ablation (RFA) is the first choice for patients with early-stage HCC [6]. However, the highly heterogeneous biological behaviour of tumour cells and liver function of patients with HCC affect the curative effect of LRT. The objective response rate of TACE is about 15–61% and 70–80% of patients who receive TACE will eventually die of tumour progression [7–10]. Patients who respond poorly to LRT need timely conversion to systemic therapy, such as sorafenib and targeted kinase inhibitor [11–13]. Therefore, it is essential to predict the outcome of LRT.

Several indicators have been shown to be related to HCC prognosis after LRT, including clinical characteristics such as alpha fetoprotein and imaging features such as tumour size, tumour numbers, the presence of nonsmoothed tumour margins, hypoattenuating halos and internal arteries [14–17]. Furthermore, several studies have shown that radiomics models with or without clinical factors can predict initial treatment response or long-term survival after LRT in HCC patients [18–22]. Nevertheless, most of the research used preoperative images which cannot provide information on the sensitivity of tumour cells to LRT. The response to initial treatment was likely influenced by operator experience. Recently, Godefroy A et al. reported that postoperative arterial phase radiomics features could predict early treatment response to ^90^yttrium transarterial radioembolization in patients with HCC [23]. Several studies have shown that texture analysis and radiomics of postoperative CT images can be used to evaluate local recurrence and tumour progression after ablation [24, 25]. However, there has been no radiomics model based on initial postoperative MRI for predicting treatment response to LRT.

The aim of this study was to investigate the role of MRI-based radiomics and nomogram for predicting the outcome of LRT at 6 months, which may contribute to guiding individualized treatment and follow-up in patients with HCC.

## Patients and methods

### Patients

**Fig. 1:**
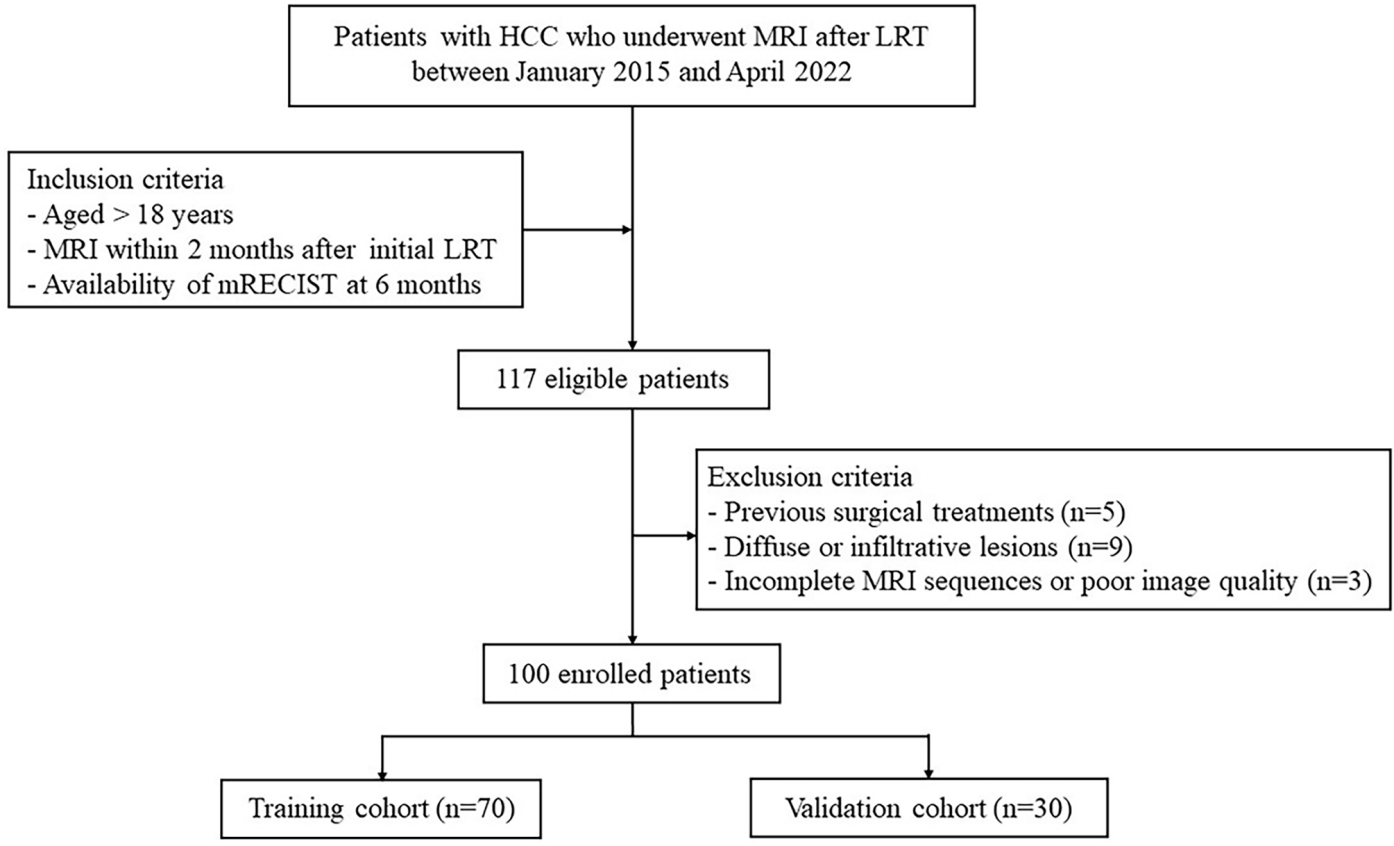
Flowchart of patient selection

This study was approved by the Institutional Review Board of our hospital and the requirement for informed consent was waived. We retrospectively collected patients who underwent LRT in our hospital between January 2015 and April 2022. Informed consent requirement was waived due to the retrospective nature of the study. The inclusion criteria were (1) presence of HCC diagnosed by pathology or typical imaging findings according to the guidelines of the American Association for the Study of Liver Disease (AASLD), (2) initial treatment was TACE or RFA, (3) aged > 18 years, (4) MRI within 2 months after initial LRT, and (5) availability of mRECIST at 6 months. The exclusion criteria were (1) previous treatments, including liver resection and transplantation (n = 5), (2) diffuse or infiltrative lesions (n = 9), and (3) incomplete MRI sequences or poor image quality (n = 3). Finally, 100 patients were included in the study and were randomly divided into the training cohort (n = 70) and the validation cohort (n = 30) at a ratio of 7:3 (Fig. 1).

Postoperative clinical characteristics of the enrolled patients, including sex, age, Barcelona Clinic Liver Cancer (BCLC) stage, history of chronic liver disease, alpha-fetoprotein (AFP), aspartate aminotransferase (AST), alanine aminotransferase (ALT), alkaline phosphatase (ALP), gamma-glutamyltranspeptidase (GGT), total bilirubin (TBIL), indirect bilirubin (IBIL), direct bilirubin (DBIL), and high-sensitivity C-reactive protein (hCRP), were collected.

### Locoregional treatment methods

TACE was performed under the guidance of digital subtraction angiography (GE Innova 3100) using the Seldinger technique. The femoral artery was punctured at the groin area and a 2.2–2.4 F microcatheter (Asahi Intecc Co. Ltd, Japan) was inserted into the feeding hepatic artery, then chemoembolization was performed. This emulsion was created using 10 ml of lipiodol (Alicon, Hanzhou, China) and 10ml of chemotherapeutic agent (with 50 mg of doxorubicin). The volume of chemoembolic emulsion injected depended on patient factors and tumor size. Following this injection, 100–300 μm gelatin sponge particles was administered to achieve the embolization end point. Percutaneous RFA was performed under the guidance of CT (GE Revolution). Two RFA systerms, Rita StarburstT Flex/talon electrode (RITA Medical Systems, Mountain View, Calif., USA), and CELON ProSurge (Olympus Winter & Ibe GmbH, Hamburg, Germany) with a deployment 2–5 cm determined by type of generator model, which were equipped with internal liquid circulation (saline solution) keeping surface temperature. The generator model selection and electrode shaft distribution were depended on the size, location and adjacent structure of the tumor. The assistance of multiplanar reformation ensured that the tip of the electrode shaft was inside or at the center of the tumor covered in expandable needles with at least 5–10 mm safety margin. Ablation-related parameters were set as per manufacturer’s instructions regarding tumor-related characteristics.

### Image acquisition

MRI examinations were obtained within 2 months after initial LRT and performed from standard institutional liver MRI protocols using 1.5-T and 3.0-T MRI scanners, including Siemens (Prisma, Magnetom), GE Healthcare (750w Discovery, HDi Signa) and Philips (Ingenia, dStream) systems. The following sequences were used: T2WI, dynamic multiphase contrast-enhanced T1WI. Detailed scanning parameters are described in Table 1. The images in the arterial phase and portal venous phase were acquired at 15–20 s and 60–70 s after the initiation of an intravenous injection of gadopentetate dimeglumine (Magnevist, Bayer Schering Pharma AG) at a dosage of 0.1 mmol/kg and a rate of 2.0 ml/s followed by a normal saline flush.

**Table 1:** MRI Scanning Parameters

	T2WI	Dynamic multiphase contrast-enhanced T1WI
Breath	Respiratory triggering	Hold breath
Fat suppression	Yes	Yes
TR (ms)	2–3 respratory cycles	3.4–4.1
TE (ms)	85	1.15–1.91
Flip angle (°)	150	15
Matrix	288 × 224	288 × 172–320 × 216
FOV (mm)	380–420	380–420
Slice thickness (mm)	6–8	3–4

### Treatment response assessment

The primary outcome was the tumour response of the target lesion at 6 months, according to mRECIST. The responses were classified as follows: (i) complete response was the disappearance of any intratumoural arterial enhancement in the target lesion; (ii) partial response was at least a 30% decrease in the sum of the longest viable tumour diameters of the target lesion; (iii) stable disease was any patient that showed neither a sufficient decrease to qualify for partial response nor a sufficient increase to qualify for progressive disease; and (iv) progressive disease was at least a 20% increase in the sum of the longest viable tumour diameters of the target lesion. For T1-weighted image hyperintense lesions, subtraction images were used to assist in the evaluation of treatment response. Objective response (OR) included complete response and partial response, whereas nonresponse (NR) included stable disease and progressive disease. The tumour response was evaluated by two radiologists (each with > 20 years of experience in hepatic imaging). The disagreements between the two radiologists were resolved through consultation.

### Radiomics analysis

To reduce the potential impact among different vendors, scanners and scanning parameters, all MRI sequences were normalized using the Z score. N4 bias correction was performed to normalize nonuniform intensity. Then, the tumour volume of interest (VOI) was manually delineated slice-by-slice on arterial phase, portal venous phase and T2-weighted images (T2WI) by a radiologist with 5 years of experience in abdominal imaging using 3D Slicer V5.0.3 (https://www.slicer.org/) software. The VOIs were checked and adjusted by a senior radiologist with 20 years of experience in abdominal imaging. Next, the VOIs were resampled into voxels of 1 × 1 × 1 mm. A total of 851 radiomics features were finally extracted from each VOI using the 3D slicer. The extracted radiomics features were divided into eight categories: 18 first-order statistics features, 14 shape-based features, 24 grey level cooccurrence matrix (GLCM) features, 14 grey level dependence matrix (GLDM) features, 16 grey level size zone matrix (GLSZM) features, 16 grey level run length matrix (GLRLM) features, 5 neighbouring grey tone difference matrix (NGTDM) features, and 744 wavelet transformed features.

The significantly different radiomics features between OR and NR in the training cohort were selected by using the independent sample t test or nonparametric Mann‒Whitney U test. Then, least absolute shrinkage and selection operator (LASSO) with penalty parameter tuning conducted by 5-fold cross-validation was further performed to identify the most valuable features. To avoid unit limits on the data of radiomics features, Z score normalization was used in the training cohort and the validation cohort. The radiomics models were constructed using the selected features via multivariate logistic regression analysis. The radiomics score (Rad score) was a linear combination cluster of the selected feature multiplied by the corresponding LASSO coefficient.

**Fig. 2:**
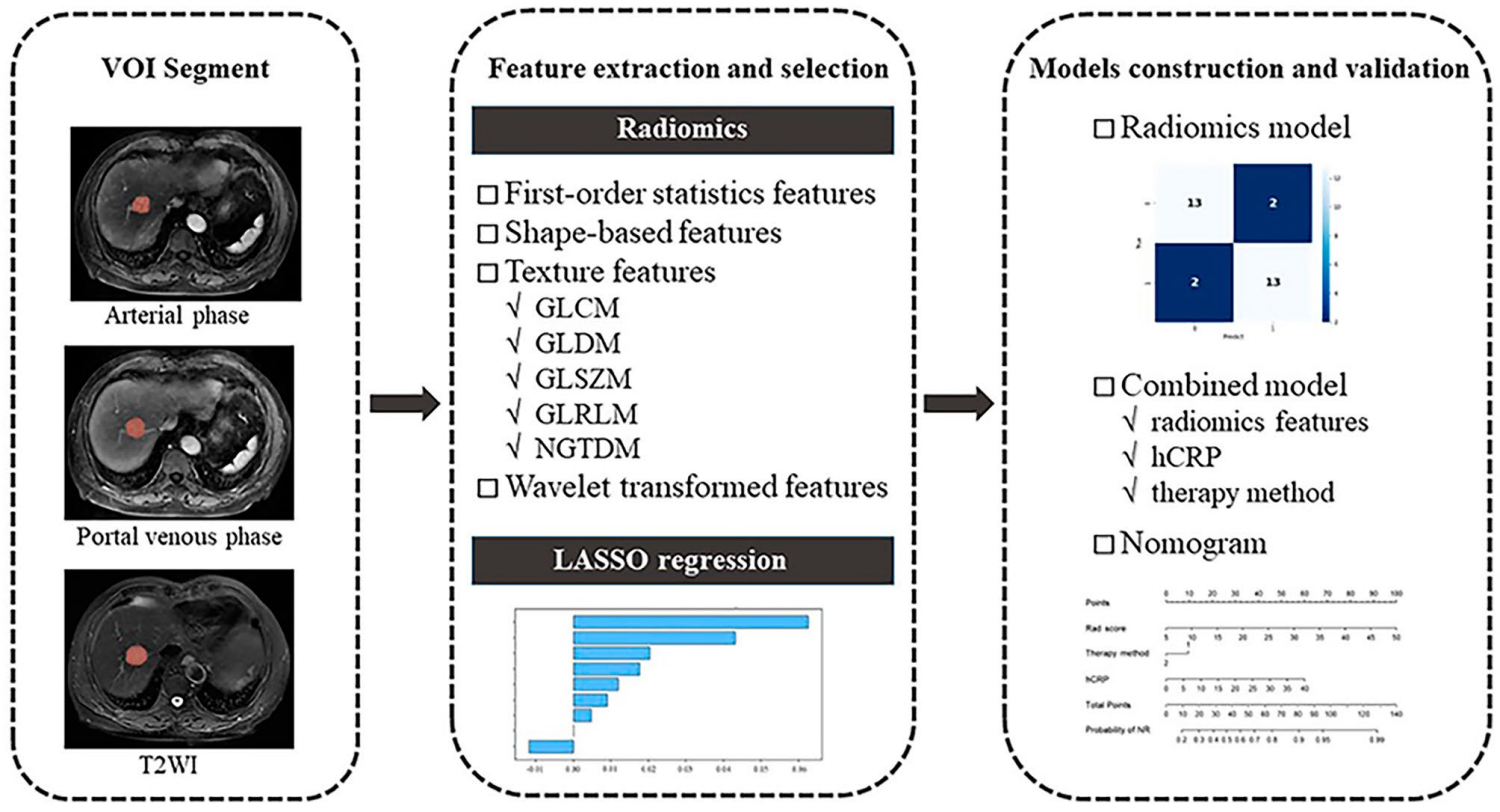
Flowchart of radiomics model and nomogram construction

The significantly different clinical variables between OR and NR in the training cohort were selected by using univariate analysis and multivariate analysis. The combined model was constructed using the above selected radiomics features and clinical variables via a multivariate logistic regression algorithm. Then, a nomogram was constructed incorporating the Rad score and selected clinical variables. The radiomics workflow is presented in Fig. 2.

### Statistical analysis

Python 3.9.12 software was used to select radiomics features and construct models. The predictive efficiency of the radiomics model and combined model was quantified by the area under the receiver operating characteristic (ROC) curve (AUC) in the validation cohort. Comparisons between the AUCs of the radiomics model and combined model were performed using Delong’s test. Calibration curves and the Hosmer‒Lemeshow test were used to evaluate the predictive efficiency of the nomogram. Continuous variables between the training cohort and the validation cohort were compared using Student’s t test or the Mann‒Whitney U test, and categorical variables were compared using the chi-squared test or Fisher’s exact test. *P* < 0.05 was considered to indicate statistically significant differences.

## Results

### Patient characteristics

In this study, 50 patients had OR, and the other 50 patients had NR. The clinical characteristics are shown in Table 2. The clinical characteristics between the training cohort and the validation cohort were balanced for there was no significant difference between the two groups.

**Table 2:** Patient clinical characteristics

	Training cohort (n = 70)	Validation cohort (n = 30)	Univariate analysis in the training cohort			Multivariate analysis in the training cohort
			OR (n = 35)	NR (n = 35)	*P* value	*P* value
Treatment response (OR:NR)	35:35	15:15	/	/	/	/
Age	63.2 ± 11.8	61.6 ± 9.3	63.3 ± 11.7	63.0 ± 12.1	0.895	
Sex (M:F)	48:22	25:5	21:14	27:8	0.126	
AFP(>15ng/ml)	26	7	10	16	0.141	
hCRP(mg/L)	2.18 (0.78 ~ 10.45)	2.14 (0.94 ~ 7.21)	1.85 (0.76 ~ 5.84)	4.14 (0.96 ~ 21.32)	**0.012**	**0.033**
ALT(> 50U/L)	10	3	5	5	1.000	
AST(> 40U/L)	19	7	9	10	0.788	
GGT(> 60U/L)	25	10	10	15	0.215	
ALP(> 125U/L)	25	10	10	15	0.215	
TBIL(> 17.1umol/L)	35	21	14	21	0.097	
DBIL(> 6.84umol/L)	21	10	7	14	0.072	
IBIL(> 12umol/L)	39	21	17	22	0.231	
With history of chronic liver disease	61	27	31	30	0.722	
BCLC (A:B)	55:15	23:7	31:4	24:11	**0.049**	
Therapy method (TACE:RFA)	46:24	23:7	18:17	28:7	**0.014**	**0.048**

### Radiomics model

**Fig. 3:**
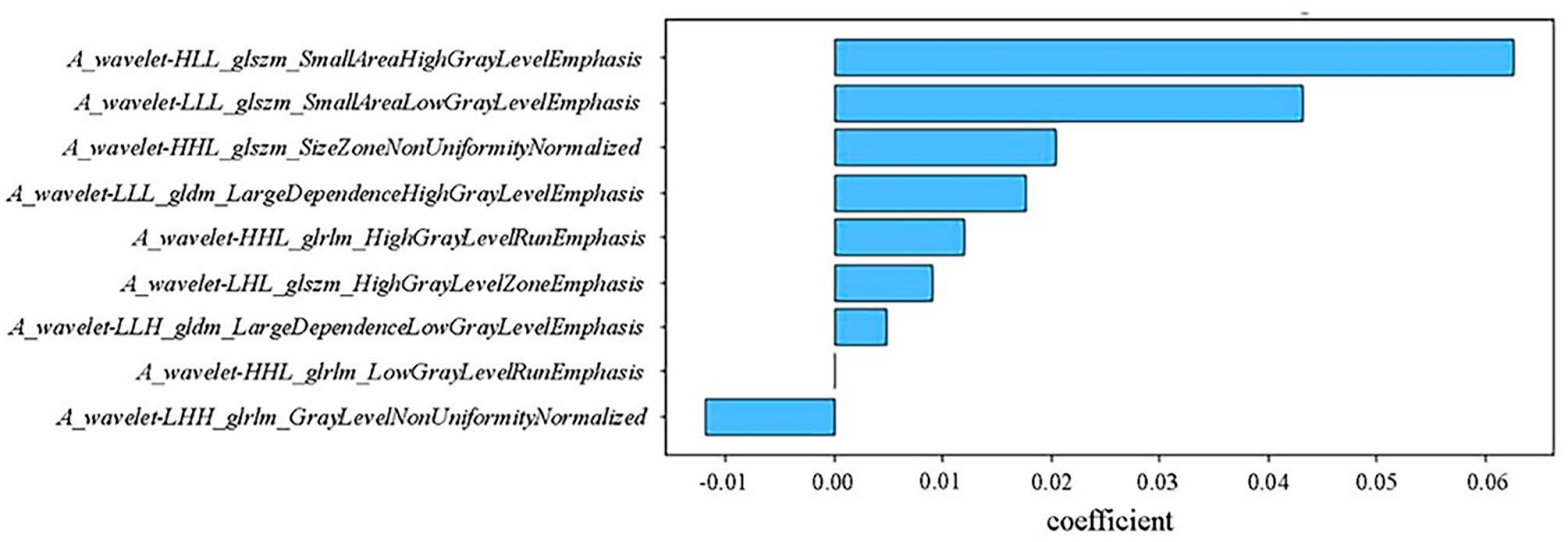
The coefficients of the selected radiomics features

Nine radiomics features in the arterial phase were selected after LASSO, and the coefficients are presented in Fig. 3. None of the portal venous phase or T2WI radiomics features were predictive of the treatment response. Nine radiomics models were built by removing the radiomics features in sequence according to the coefficients. The best radiomics model with the first five radiomics features showed the highest AUC of 0.833 (95% CI, 0.653–0.9440 in the validation cohort.

### Combined model

**Fig. 4:**
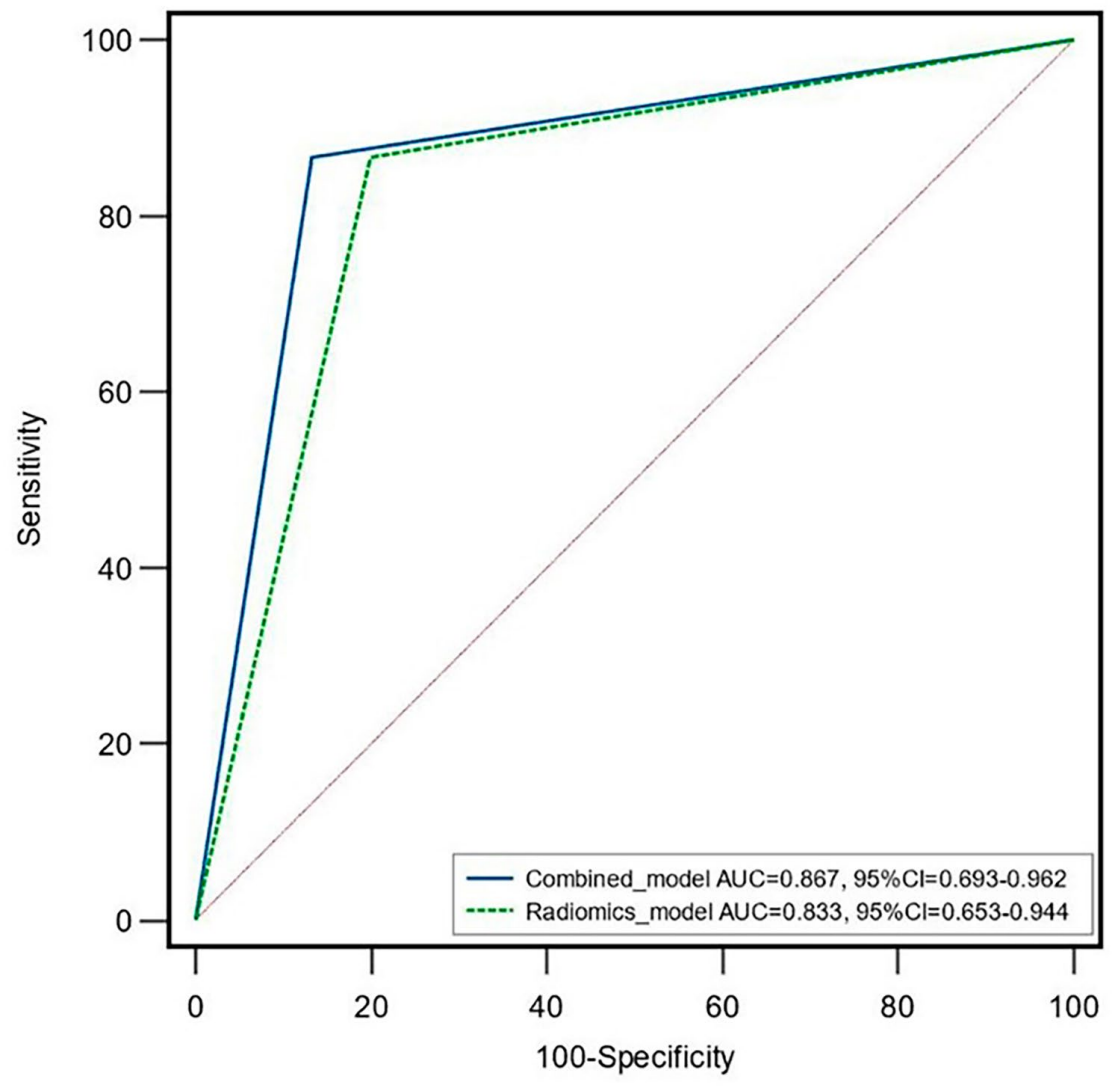
ROC curves of the radiomics model and combined model

Two clinical variables (hCRP and therapy method) were selected through the univariate analysis and multivariate analysis (Table 2). A combined model was built incorporating the best five radiomics features and the two clinical variables. The AUC of the combined model was 0.867 (95% CI, 0.693–0.962) in the validation cohort. However, there was no significant difference in the AUC between the combined model and the best radiomics model (*P* = 0.573), as shown in Fig. 4. The distinguishing efficiency of the two models is shown in Table 3.

**Table 3:** Distinguishing efficiency of the two models

	Sensitivity	Specificity	Accuracy	AUC
Best radiomics model	0.87	0.80	0.83	0.833
Combined model	0.87	0.87	0.87	0.867

### Nomogram

**Fig. 5:**
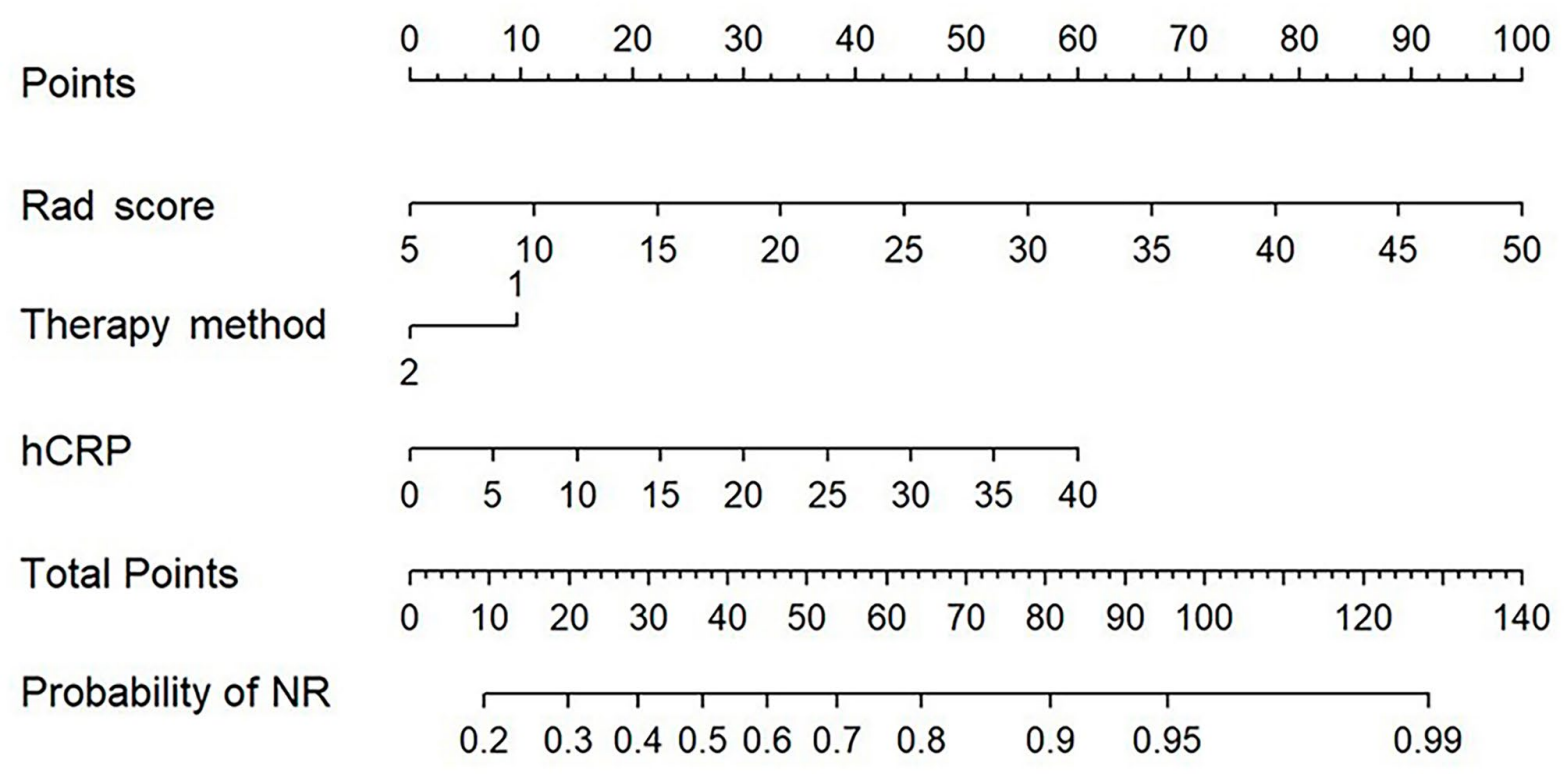
Nomogram based on Rad score, therapy method and hCRP The probability of NR for each patient is marked on the axis

**Fig. 6:**
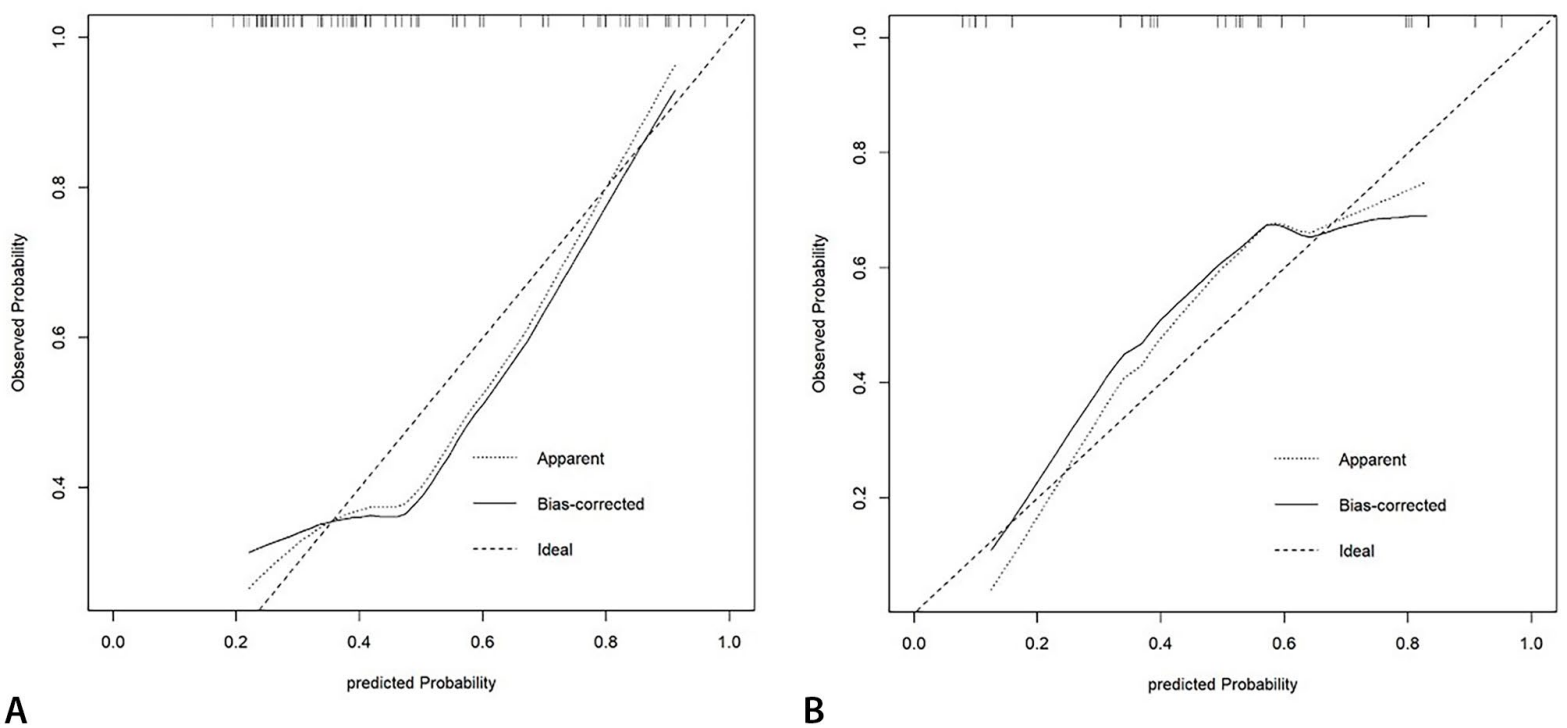
The calibration curves in the training cohort (A) and the validation cohort (B) The diagonal dotted line represents the ideal evaluation, while the solid lines and dashed lines represent the performance of the corrected and apparent bias, respectively. The prediction solid line is close to the ideal dotted line, meaning that the model has good prediction accuracy

The nomogram was constructed with the Rad score and selected two clinical variables to individually predict tumour response (Fig. 5). The calibration curves demonstrated good agreement between the prediction and the observation in both cohorts (Fig. 6). The Hosmer‒Lemeshow test showed nonsignificant result suggesting a satisfying fit of the nomogram.

## Discussion

The present study constructed a radiomics model and a combined model for predicting the outcome of LRT at 6 months in HCC patients, and the models had AUCs of 0.833 and 0.867, respectively, in the validation cohort. Also, a nomogram was established based on Rad scores and clinical variables and could stratify patients into OR and NR groups. To the best of our knowledge, this is the first study using a radiomics model based on initial postoperative MRI to predict the treatment response of HCC patients undergoing LRT.

Previous studies have shown that radiomics models with or without clinical factors had good performance in predicting the first TACE response in patients with HCC (AUC 0.815-0.900) [26–28]. We assessed the treatment response at 6 months, which possessed more clinical value than that after the first treatment. Recent report used MRI radiomics nomogram to predict recurrence after ablation at 1, 2, and 3 years in HCC patients, the AUCs of which (0.72, 0.61 and 0.64) were lower than our study (0.867) [29]. Moreover, most published studies used CT-based radiomics [21, 22, 24, 26–28, 30], but MRI is known to have higher soft-tissue contrast than CT and is more commonly used in treated HCC viability evaluation. Based on the reports that postoperative CT and MRI were of predictive value for outcome of LRT [23–25], our study used initial postoperative MRI.

The patients in our study at BCLC B or A stage received TACE or RFA, reflecting the real phenomenon in the clinical setting. There was no significant difference in BCLC stage between OR and NR in the training cohort after the multivariate analysis. Therapy method showed significant difference and was put into combined model. Nevertheless, the radiomics model without regard to the therapy method showed satisfactory predictive ability, and the AUC of the combined model was higher than that of the radiomics model without significant difference. Our study enrolled patients receiving TACE or RFA, which attached the model wider clinical application.

Our study used MRI-based radiomics and performed normalization to correct the scanner and individual effect. In addition, no radiomics feature in the portal venous phase and T2WI was selected after LASSO, consistent with clinical experience that viability evaluation mainly relies on the arterial phase; thus, we established the radiomics model based on only the arterial phase, which is simple and convenient in clinical practice. A previous study also indicated that only postoperative arterial phase radiomics features were predictors for treatment response in patients with HCC [24] and supported our findings. We obtained the best radiomics model using the first five of the nine selected radiomics features, consistent with the literature that the number of selected radiomics features should be less than 10% of the total number of positive cases [31].

To improve general applicability, we constructed a nomogram based on the Rad score, therapy method and hCRP. Calibration curves demonstrated favourable prediction performance of our nomogram. Several studies have shown that hCRP was marker of poor prognosis in patients with HCC [32, 33]. However, unlike previous literature, we found that AFP had no relationship with treatment response at 6 months. This may be because AFP in our study was collected after initial treatment.

This study has several limitations. First, this was a single-centre retrospective study, the sample size of patients was relatively small, and the study results were without external validation. A much larger database of prospective studies will be collected from more centres in the future. Second, we used MRI machines with different Tesla intensity (1.5 vs. 3T), which could reduce the accuracy of our model. Third, delineating the VOIs mostly depended on the radiologists’ experience, and the manual method required considerable time and energy. Future studies could develop an automatic segmentation model for focal liver lesions to minimize discrepancies. Last, the biological mechanisms resulting in these radiomics features are still unknown. Multiomics, including radiomics, genetics and proteomics, will become the focus of future research.

## Conclusion

In conclusion, radiomics features derived from initial postoperative MRI may be potential biomarkers for predicting tumour response to LRT at 6 months. The nomogram with the Rad score and clinical variables demonstrated favourable prediction performance and general applicability which may aid in further treatment planning in patients with HCC.

## Data Availability

The datasets used and analysed in the current study are available from the corresponding author on reasonable request.

## Abbreviations

HCC: Hepatocellular carcinoma
LRT: Locoregional treatment
TACE: Transcatheter arterial chemoembolization
RFA: Radiofrequency ablation
OR: Objective response
NR: Nonresponse
Rad score: Radiomics score
